# Extravasated platelet aggregation in liver zone 3 may correlate with the progression of sinusoidal obstruction syndrome following living donor liver transplantation: A case report

**DOI:** 10.3892/etm.2015.2245

**Published:** 2015-02-02

**Authors:** SHINICHI NAKANUMA, TOMOHARU MIYASHITA, HIRONORI HAYASHI, HIDEHIRO TAJIMA, HIROYUKI TAKAMURA, TOMOYA TSUKADA, KOICHI OKAMOTO, SEISHO SAKAI, ISAMU MAKINO, JUN KINOSHITA, KEISHI NAKAMURA, KATSUNOBU OYAMA, MASAFUMI INOKUCHI, HISATOSHI NAKAGAWARA, ITASU NINOMIYA, HIROHISA KITAGAWA, SACHIO FUSHIDA, TAKASHI FUJIMURA, TETSUO OHTA

**Affiliations:** Department of Gastroenterologic Surgery, Division of Cancer Medicine, Graduate School of Medical Science, Kanazawa University, Kanazawa, Ishikawa 920-8641, Japan

**Keywords:** platelet, aggregation, cluster of differentiation 42b, sinusoidal obstruction syndrome, veno-occlusive disease, liver transplantation

## Abstract

Sinusoidal obstruction syndrome (SOS), previously known as veno-occlusive disease, is relatively rare subsequent to liver transplantation (LT). SOS refractory to medical therapy, however, can result in centrilobular fibrosis, portal hypertension and liver failure. Although sinusoidal endothelial cell damage around central venules (zone 3) occurs early in the development of SOS, the detailed mechanism of SOS development and its association with thrombocytopenia are not yet completely understood. The present report describes a patient who experienced SOS with unexplained thrombocytopenia following living donor LT. The progression of SOS resulted in graft dysfunction and the patient succumbed. The presence of platelets in the liver allograft was assayed immunohistochemically using antibody to the platelet marker cluster of differentiation 42b (platelet glycoprotein Ib). Platelet aggregates were found attached to hepatocytes along the sinusoid and within the cytoplasm of hepatocytes, particularly in zone 3. By contrast, no staining was observed in zone 1. These findings suggested that extravasated platelet aggregation in the space of Disse and the phagocytosis of platelets by hepatocytes were initiated by sinusoidal endothelial cell damage due to the toxicity of the immunosuppressant tacrolimus or a corticosteroid pulse, and that platelet activation and degranulation may be at least partially involved in the mechanism responsible for SOS.

## Introduction

Sinusoidal obstruction syndrome (SOS), a condition previously known as veno-occlusive disease (1), is a life-threatening syndrome that results from sinusoidal congestion and is characterized by hepatomegaly, ascites, portal hypertension, weight gain and jaundice (2). This syndrome is commonly observed as a complication of high-dose chemotherapy administered prior to hematopoietic progenitor cell (HPC) transplantation (3). The development of SOS subsequent to liver transplantation (LT) is relatively uncommon, being observed in ~2% of LT recipients (4). SOS is severe and refractory to medical therapy, with the ultimate solution being re-transplantation (2).

The mechanism underlying the development of SOS is not yet completely understood, although sinusoidal endothelial cell damage leading to alterations in the hemostatic system is regarded as central to the pathogenesis of SOS (5). SOS has additionally been reported to be associated with platelet functions. For example, transforming growth factor (TGF) β-1 secreted by platelets has been shown to contribute to hypercoagulability in SOS following HPC transplantation (6). In addition, plasminogen activator inhibitor-1 (PAI-1), which is abundant in platelets, has been suggested to be a specific marker of SOS (7). Clinically, patients who experience SOS following HPC transplantation are both thrombocytopenic and refractory to platelet transfusions (8). These observations suggest that platelets may be important in the pathogenesis of SOS.

The present case report describes a patient who experienced liver allograft dysfunction caused by the progression of SOS following living donor LT (LDLT), with unexplained thrombocytopenia occurring around the same period of time. We hypothesized that the platelets had been consumed by the liver allograft, and therefore the allograft was immunohistochemically assayed for the presence of platelets using antibody to the platelet marker cluster of differentiation (CD)42b (platelet glycoprotein Ib).

## Case report

A 59-year-old male patient was diagnosed with primary biliary cirrhosis-autoimmune hepatitis overlap syndrome and end-stage cirrhosis. His Child-Pugh score was C 12 points and his Model For End-Stage Liver Disease score was 23 (9). The donor was the patient’s 56-year-old wife. Their blood types were compatible (blood type O→AB), and the lymphocyte cross-match test was negative. The LDLT involved the right lobe without the middle hepatic vein (500 g). The graft-to-recipient weight ratio was 0.83, with the graft constituting 42% of the standard liver volume of the recipient. Following transplantation, the patient was started on immunosuppressive treatment with tacrolimus (Tac), mycophenolate mofetil (MMF) and prednisolone. His immediate postoperative course was good, and he was moved from the intensive-care unit on postoperative day (POD) 3.

**Figure 1:**
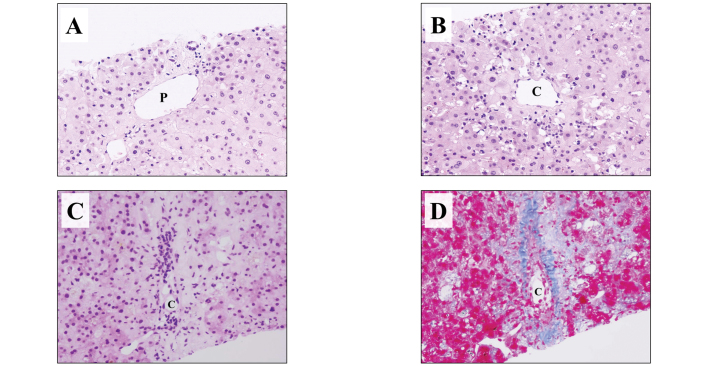
Histopathology of the liver biopsy sample obtained on POD 91 and 203 (magnification, ×200). (A and B) Samples taken on POD 91 showing (A) minimal inflammation and no bile duct injury in the portal area and (B) chronic inflammation and loss of hepatocytes in zone 3 (hematoxylin and eosin staining). (C and D) Samples taken on POD 203 showing (C) inflammatory cells infiltrating into the liver parenchyma, severe loss of hepatocytes and central vein obliteration (hematoxylin and eosion staining) and (D) basement membrane formation caused by fibrous tissue and sinusoidal fibrosis, with progressive worsening of the central perivenulitis and the development of sinusoidal obstruction syndrome (Masson’s trichrome stain). C, central vein; P, portal vein; POD, postoperative day.

Approximately 2 months after the LDLT, the patient’s concentrations of biliary tract enzymes and total bilirubin (T-Bil) began to increase progressively. Endoscopic retrograde cholangiopancreatography revealed no stenosis of the biliary anastomosis, and color doppler ultrasonography and computed tomography revealed no abnormalities. A liver biopsy on POD 77 showed minimal inflammation of the portal area and slight endothelial inflammation of the central venules. At that time, the patient’s platelet counts were <10×10^5^/mm^3^ and his T-Bil concentration continued to increase, to 17 mg/dl. The patient was regarded as experiencing late-onset acute rejection and was treated with augmented immunosuppressive therapy, consisting of intravenous corticosteroid pulse therapy, increased doses of MMF and adjustment of the Tac trough level to maintain a whole-blood concentration of 5–10 ng/ml. A liver biopsy taken on POD 91 showed minimal inflammation and no bile duct injury in the portal area (Fig. 1A), similar to the previous biopsy. Zone 3, however, showed chronic inflammation and a loss of hepatocytes (Fig. 1B). The sample was negative for C4d staining, a marker of antibody-mediated rejection. Based on these findings, the patient was diagnosed with late-onset acute rejection accompanied by mild central perivenulitis, resulting in an intensification of immunosuppression. Since the concentration of Tac could not be maintained >10 ng/ml, despite increased doses, the patient’s calcineurin inhibitor treatment was switched from Tac to cyclosporin A (CsA), with the CsA dose adjusted to maintain a target level in the blood of 150–200 ng/ml, and everolimus was added as a third immunosuppressive agent. In addition, the patient was administered two doses of basiliximab (50 mg/day) to suppress the antibody-mediated rejection. The patient’s platelet counts declined to ~5×10^5^/mm^3^, despite the lack of any apparent causes of the thrombocytopenia, such as disseminated intravascular coagulation, thrombotic microangiopathy or any other thrombotic diseases; however, his T-Bil concentration increased to 21.4 mg/dl. A liver biopsy taken on POD 203 showed inflammatory cells infiltrating the liver parenchyma in zone 3, as well as marked reductions in hepatocytes and central vein obliteration, as revealed by hematoxylin and eosin staining (Fig. 1C). Masson’s trichrome stain showed basement membrane formation caused by fibrous tissue and sinusoidal fibrosis (Fig. 1D). By contrast, the portal area (zone 1) showed minimal inflammation, similar to the previous biopsy. The patient’s central perivenulitis had progressively worsened, resulting in the development of SOS. His platelet count was ~5×10^5^/mm^3^, the allograft was less responsive to augmented immunosuppressive therapy and his condition was considered irreversible. Re-transplantation was indicated if feasible, but a donor could not be found. He subsequently experienced liver and renal failure and succumbed on POD 250.

### Immunohistochemistry of CD42b

**Figure 2:**
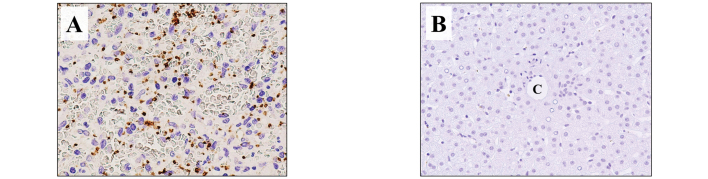
Immunohistochemical staining of (A) normal spleen and (B) normal liver with antibody to cluster of differentiation 42b. (A) Immunoreactivity was evident throughout the spleen as dots, which were morphologically characterized as platelets (magnification, ×400). (B) No staining of normal liver tissue was observed (magnification, ×200). C, central vein.

**Figure 3:**
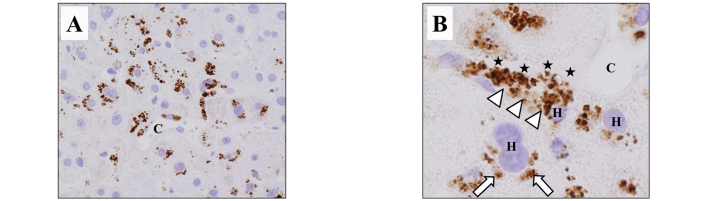
Immunohistochemical staining of liver allograft tissues with antibody to CD42b. (A) The liver biopsy taken on postoperative day 91 showed CD42b expression in zone 3 (magnification, ×400). (B) At higher magnification, CD42b was present as aggregates attached to hepatocytes (arrowheads) along the sinusoid (★) and in the hepatocyte cytoplasm (arrows) (magnification, ×1,000). C, central vein; H, hepatocyte; CD42b, cluster of differentiation 42b.

The presence of platelets in the liver tissue samples was assayed using a mouse monoclonal antibody to CD42b (1:100; cat. no. EPR6995; Abcam, Tokyo, Japan), a marker expressed on both inactive and activated platelets. In normal spleen tissue, used as a positive control, CD42b expression was evident as dots, morphologically characterized as platelets (Fig. 2A), whereas there was no staining of normal liver tissue, used as the negative control (Fig. 2B). Assay of the liver allograft tissue obtained from the patient on POD 91 showed CD42b expression in zone 3 (Fig. 3A). At greater magnification, the CD42b appeared as aggregates attached to hepatocytes along the sinusoids. CD42b was also expressed in the cytoplasm of the hepatocytes (Fig. 3B) but was not observed in zone 1.

## Discussion

SOS is believed to result from sinusoidal endothelial cell damage in zone 3 of the liver (5). Red blood cells subsequently leak into the space of Disse, depositing fibrin (1). The basement membrane that forms from the fibrous tissue leads to hepatocyte ischemia, followed by hepatocyte necrosis (1). Tac administration following transplantation may be due to sinusoidal endothelial cell damage of the small hepatic vein in zone 3 (10,11). To date, however, the pathophysiology of SOS remains incompletely understood, with no definitive mechanisms or effective treatments identified.

There are few reports concerning a pathologic association between platelets and SOS. One study, in which liver samples taken at autopsy from HPC transplant recipients with SOS were stained with an anti-platelet antibody, found no evidence of platelet deposition (12); however, to prevent SOS, its pathogenetic mechanism must be clarified prior to it becoming irreversible. The present study therefore assessed liver tissue samples taken from an LDLT recipient in the development process of SOS. Zone 3 of the liver was positive for CD42b, with the staining pattern observed as aggregates attached to hepatocytes along the sinusoid and in the cytoplasm of the hepatocytes. These findings suggest that extravasated platelet aggregation (EPA) was present in the space of Disse, and that the platelets were phagocytized by hepatocytes. It is likely that these aggregated and activated platelets in the space of Disse had a greater influence on hepatocytes than did the platelets in the sinusoidal vessels.

**Figure 4:**
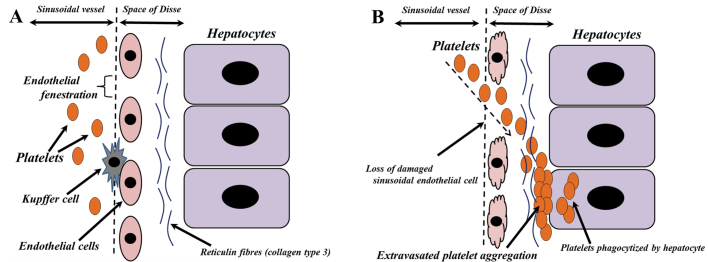
Schematic model of (A) a normal sinusoid and (B) the sinusoid of sinusoidal obstruction syndrome in the patient in the present study. The normal sinusoid is a type of sinusoidal blood vessel with endothelial fenestrations (A). Damage to the sinusoidal endothelium can result in the denuding of the endothelium or the loss of fenestrations, allowing platelets to enter the space of Disse. This space contains reticulin fibers, which consist primarily of collagen type 3. Platelets can easily attach to collagen type 3, forming aggregates, including in the space of Disse. In addition, the extravasated platelets in the space of Disse can be phagocytized by hepatocytes through the asialoglycoprotein receptor (B).

Liver sinusoids are a type of sinusoidal blood vessel with endothelial fenestrations (13) (Fig. 4A), the diameters of which are increased by sinusoidal endothelial cell damage (14). The sinusoidal endothelial cells in the patient described in the present report had been exposed to Tac or a corticosteroid pulse, resulting in sinusoidal endothelial cell damage. This damage likely contributed to the denuding of the endothelium or loss of the fenestrations, allowing platelets to enter the space of Disse (Fig. 4B). This space contains reticulin fibers, most of which contain collagen type 3 (15). Platelets have been found to bind to and form aggregates with collagen type 3 (16), resulting in platelet aggregation in the space of Disse (Fig. 4B).

Hepatocytes contribute to blood homeostasis by endocytosing a number of proteins present in plasma (17). The asialoglycoprotein receptor (ASGR), a membrane-bound lectin, removes the target glycoproteins from the circulation (18). These glycoproteins are also found on the surface of platelets, and human platelets have been reported to be sequestered by hepatocytes through the ASGR (19). In the patient in the present report, the extravasated platelets in the space of Disse may have been phagocytized by hepatocytes through the ASGR (Fig. 4B).

**Figure 5:**
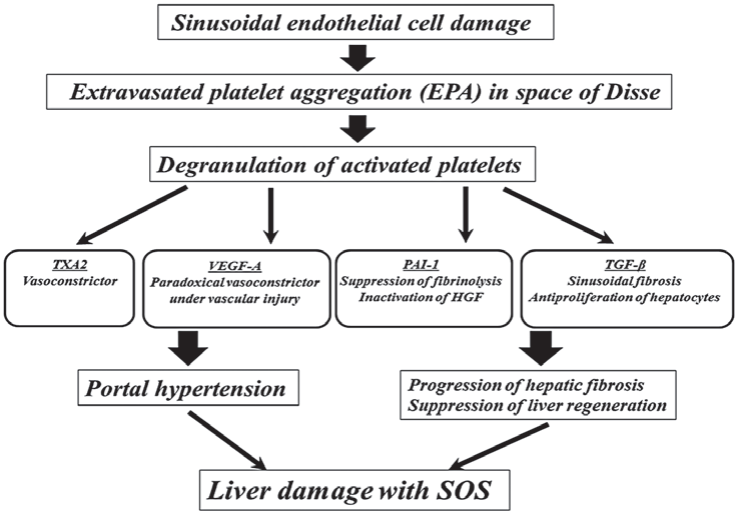
The predicted pathogenic mechanism of SOS in the patient in the present study. Extravasated platelet aggregation in the space of Disse was initiated by damage to the sinusoidal endothelium induced by tacrolimus or a corticosteroid pulse. The negative regulators released by activated platelets, including TXA2, VEGF-A, PAI-1 and TGF-β, may have induced portal hypertension and the progression of hepatic fibrosis, as well as suppressed liver regeneration, initiating liver damage with SOS. SOS, sinusoidal obstruction syndrome; TXA2, thromboxane A2; VEGF-A, vascular endothelial growth factor-A; PAI-1, plasminogen activator inhibitor-1; HGF, hepatocyte growth factor; TGF-β, transforming growth factor-β.

Platelets contain α and dense granules (20). Upon activation, platelets excrete the contents of these granules into their canalicular systems or the surrounding blood (20). These granules also contain negative regulators of liver regeneration, including thromboxane A2 (TXA2), vascular endothelial growth factor-A (VEGF-A), TGF-β and PAI-1 (6,21,22). TXA2 is a vasoconstrictor that increases portal venous resistance (23) and causes portal hypertension. Although VEGF-A acts as a vasodilator under ordinary circumstances, it acts, paradoxically, as a vasoconstrictor in patients with endothelial failure (24). Bevacizumab, an antibody against VEGF-A, protects against liver injury associated with SOS (25). PAI-1 suppresses fibrinolysis and the progression to fibrosis in the tissue microenvironment. In addition, PAI-1 acts as a negative regulator of hepatocyte proliferation by inhibiting urokinase-type plasminogen activator, which activates hepatocyte growth factor (26,27). TGF-β is a major antiproliferative factor for hepatocytes (28) that stimulates collagen synthesis through activated hepatic stellate cells (29). In the patient in the present report, the release by platelets of these negative regulators may have induced portal hypertension and the progression of hepatic fibrosis, as well as the suppression liver regeneration, a mechanism responsible, at least in part, for the initiation of liver damage with SOS (Fig. 5).

No standard method of treating SOS has yet been established. Systemic anticoagulation and thrombolytic therapies have been tested extensively (30). Defibrotide, a polydeoxyribonucleic acid, has been recently shown to have a promising response rate in patients with severe SOS (31). When medical treatment fails, transjugular intrahepatic portosystemic shunt placement or re-transplantation can be considered (2). Depending on the extent of the sinusoidal endothelium damage and EPA in the space of Disse, the prophylactic administration of endothelial protective and antiplatelet agents may be effective prior to the development of irreversible damage. Cilostazol, a phosphodiesterase 3 (PDE3) inhibitor, may be appropriate, owing to its antiplatelet properties, its ability to increase tolerance to ischemia/reperfusion injury (32) and its induction of immune tolerance via an enhanced regulatory T-cell response (33). In clinical practice, we have administered a PDE3 inhibitor to LDLT recipients, with favorable outcomes (Nakanuma *et al*, unpublished data).

In conclusion, the present findings suggest that EPA in the space of Disse, which was initiated by sinusoidal endothelial cell damage due to the toxicity of Tac or a corticosteroid pulse and the negative regulators released by activated platelets, may have partially contributed to liver damage with SOS in the patient. Endothelial protective therapy or antiplatelet treatment may have been useful in the treatment of SOS in this patient. Studies in additional patients are necessary to elucidate the role of EPA in the space of Disse on the development of SOS.
